# Serum anti-Müllerian hormone levels are associated with early miscarriage in the IVF/ICSI fresh cycle

**DOI:** 10.1186/s12884-022-04591-5

**Published:** 2022-04-02

**Authors:** Xin Liu, Ying Han, Xinyan Wang, Yunshan Zhang, Aijun Du, Ruqiang Yao, Jiabei Lv, Haining Luo

**Affiliations:** Centre for Reproductive Medicine, Tianjin Central Hospital of Gynecology Obstetrics, No. 156 Nankai San Ma Road, Tianjin, 300100 China

**Keywords:** Anti-Müllerian hormone, Early miscarriage, Pregnancy rate, IVF/ICSI

## Abstract

**Background:**

Anti-Müllerian hormone (AMH) is used as a biomarker to estimate ovarian reserve. The relationship between AMH and early miscarriage of in vitro fertilization (IVF) is still inconclusive. This study aimed to explore whether serum AMH levels are associated with early miscarriage rates after in vitro fertilization/intracytoplasmic sperm injection (IVF/ICSI) with fresh embryo transfer (ET).

**Methods:**

This retrospective cohort study included 2246 patients undergoing their first oocyte retrievals for IVF/ICSI with fresh embryos transferred to Tianjin Central Hospital of Gynecology Obstetrics between May 2018 and March 2020. The serum AMH levels of the patients were measured within 12 months before the IVF/ICSI cycles. All women were divided into a low-AMH group, medium-AMH group and high-AMH group. Binary logistic regression was applied to confirm whether the serum AMH level was associated with the risk of early miscarriage independent of potential confounders, such as age, body mass index (BMI), duration of infertility, main diagnosis, history of internal medicine diseases, number of oocytes retrieved and high-quality embryo rate.

**Results:**

The early miscarriage rate was significantly lower in the medium-AMH group than in either the low-AMH or high-AMH group among young (< 35 years) women (*P* = 0.015). In women above 35 years of age, the early miscarriage rates in the three AMH groups were not significantly different. Young women with high serum AMH levels had a significantly higher risk of early miscarriage regardless of age or other potential confounders (adjusted odds ratio (OR) 2.382, 95% confidence interval (CI) 1.246 to 4.553, *P* = 0.009). The results remained similar after restricting the analysis to women without polycystic ovary syndrome (PCOS).

**Conclusions:**

With a high AMH level, young women had a higher risk of early miscarriage than women with a medium AMH level in their first IVF/ICSI treatment. In young women, serum AMH levels were independently associated with the risk of early miscarriage after IVF-ET treatment. Serum AMH levels might be a valuable marker to estimate the risk of early miscarriage. It is worth noting to the clinical value of AMH.

## Introduction

Currently, due to the pursuit of higher education and increasing pressures involving life and work, female age at pregnancy has increased gradually. With increasing age, female fertility decreases [1], and the risk of miscarriage increases [2]. It is estimated that 23 million miscarriages occur worldwide every year [3], and miscarriage affects 1 in 10 women in their lifetime.

Diminished ovarian reserve is closely related to age [4], but it may also occur in young women [5]. In recent years, anti-Müllerian hormone (AMH) has been used as a biomarker to estimate ovarian reserve and predict ovarian response [6–9]. Ovarian reserve is an important factor in predicting in vitro fertilization (IVF) outcomes [10]. As AMH has been widely accepted as a predictor of ovarian response, it seems reasonable that AMH could have some associations with IVF outcomes.

However, whether AMH plays a role in predicting the pregnancy outcome of IVF is still inconclusive. Several studies have reported that serum AMH can predict pregnancy outcomes, both spontaneous and after assisted reproductive technology [11–13], such as the live birth rate and ongoing pregnancy rate. On the other hand, some studies did not find a significant correlation between AMH and pregnancy outcomes in IVF cycles [7, 14]. While early pregnancy loss usually results from foetal aneuploidy [15], most chromosome abnormalities arise from meiosis of the oocyte [16]. Thus, we hypothesized that the prevalence of early miscarriage could indirectly provide some information on oocyte quality.

Tarasconi et al. [17] showed that miscarriage rates were significantly higher among women with low (0.08–1.60 ng/mL) serum AMH levels than among those with medium (1.61–5.59 ng/mL) or high (5.60–35.00 ng/mL) AMH levels regardless of age or number of oocytes retrieved. Lekamge et al. [18] also found that low-AMH patients had a higher risk of miscarriage during fresh embryo transfers. Hong et al. [19] reported in their multivariable logistic regression analysis that the probability of early pregnancy loss was significantly affected by age (adjusted odds ratio (OR) 1.709, 95% confidence interval (CI) 1.025 to 1.135, *P* = 0.004) and AMH (adjusted OR 0.885, 95% CI 0.797 to 0.982, *P* = 0.022). Morales et al. [20] found that serum AMH levels correlated with embryo quality on Day 5, but found no association between AMH and embryo quality on Day 3. As the possible correlation between AMH and the reproductive potential of the oocyte needs more exploration, it may be worthwhile to analyse the association between AMH and early miscarriage rates rather than focusing only on pregnancy and live birth rates. Therefore, in our study, we aimed to investigate whether serum AMH could affect the process of embryo formation and the pregnancy outcomes of IVF treatment, especially early miscarriage.

## Materials and methods

### Patients and study design

This retrospective study comprised 2246 infertile women who underwent their first in vitro fertilization/intracytoplasmic sperm injection (IVF/ICSI) treatment in the reproductive centre of Tianjin Central Hospital of Gynecology Obstetrics between May 2018 and March 2020. The inclusion criteria were as follows: 1) the woman’s first oocyte retrieval cycle for IVF/ICSI treatment with at least one D3 ET in a fresh cycle; 2) measurement of serum AMH in the 12 months prior to ovarian stimulation; 3) use of only the couples’ own gametes in the treatment; and 4) at low risk of venous thromboembolism (VTE) according to the Padua Prediction Score. The exclusion criteria were as follows: 1) women with uterine malformations that could affect embryo implantation or development; 2) couples who had chromosomal abnormalities; 3) cycles in which mature oocytes or available embryos were not acquired; or 4) cycles cancelled due to social or personal reasons.

### AMH measurement

Serum AMH levels were measured with an enzyme immunoassay kit (YHLO, Shenzhen, China). The limit of detection (LoD) was 0.06 ng/ml, and the intra- and interassay coefficients of variation (CVs%) were ≤ 15%.

### Treatment protocol

The women were treated with different ovarian stimulation protocols, including the gonadotrophin-releasing hormone (GnRH) agonist long protocol, GnRH antagonist protocol and natural cycle. The initial gonadotropin doses were dependent on the patients’ overall condition, such as age, body mass index (BMI), and ovarian reserve. The subsequent doses were adjusted in terms of ovarian response. When the diameters of 2–3 follicles reached ≥ 18 mm, the patients were injected with 2000–10,000 IU hCG or recombinant hCG. Thirty-six hours later, oocyte retrieval was carried out. One or two embryos were transferred 3 days after oocyte retrieval. The luteal phase was supported from the day of oocyte retrieval to 14 days later.

### Outcome assessment

A clinical pregnancy was confirmed when ≥ 1 intrauterine or extrauterine gestational sac was evident on transvaginal ultrasound examination 4 weeks after ET [21]. A biochemical pregnancy was defined as a serum hCG concentration > 25 IU/L 14 days after ET followed by a continuous decrease in hCG levels to negativity for hCG. An early miscarriage was defined as a spontaneous abortion that occurred within 12 weeks of amenorrhea in a clinical intrauterine pregnancy detected by ultrasound. Live birth was defined as a pregnancy that ended with the birth of a live infant.

### The division of age and AMH groups

We analysed the possible associations with serum AMH levels and pregnancy outcomes. Due to the influence of age on serum AMH levels and pregnancy outcomes, patients were first divided into two groups according to reproductive age: the young-age group (≤ 35 years, *n* = 1531) and the advanced-age group (> 35 years, *n* = 715). In addition, according to the serum AMH level, patients were divided into three groups per the 25th and 75th percentiles of the study population: low AMH (0.06–1.60 ng/ml), medium AMH (1.61–3.98 ng/ml) and high AMH (3.99–20.20 ng/ml).

### Statistical analysis

The differences in baseline characteristics, treatment information and IVF-ET outcomes between different age and AMH groups were analysed by means of the chi-square test. As the continuous data were not normally distributed, the differences in the continuous variables were analysed by the Kruskal–Wallis test.

To evaluate the impact of AMH levels on IVF-ET outcomes, the OR and 95% CI were estimated using logistic regression. The potential confounders adjusted in the multivariate analyses included maternal age, BMI, duration of infertility, main infertility diagnosis, history of internal medicine diseases (diabetes mellitus, thyroid disease, hypertension and hyperprolactinemia), number of oocytes retrieved, and high-quality embryo rate. Since polycystic ovary syndrome (PCOS) may be associated with higher rates of miscarriage, we conducted subanalyses among women without PCOS to explore the exact AMH effect on IVF-ET outcomes.

Data analysis was carried out using SPSS (version 23.0). *P* < 0.05 was considered to be statistically significant.

## Results

**Fig. 1:**
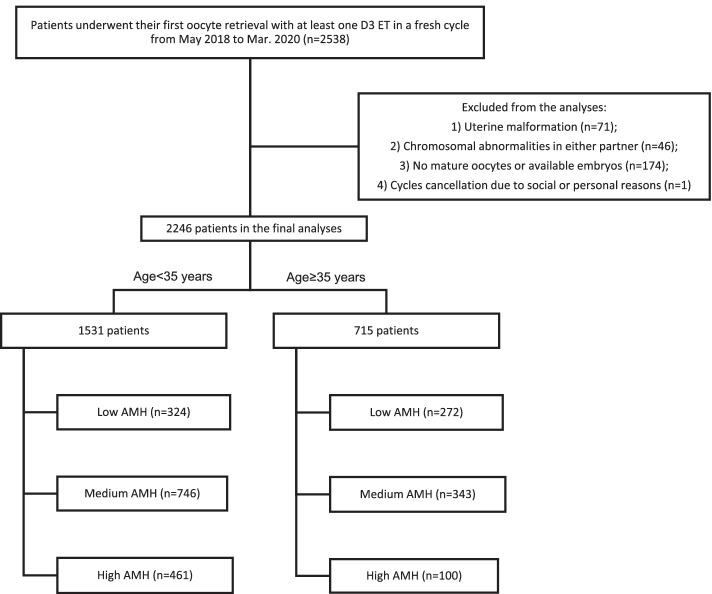
A flowchart of the patient selection process and division of the age and AMH groups

The flowchart of the study is shown in Fig. 1. A total of 2246 women who underwent their first IVF/ICSI treatment with at least one ET in a fresh cycle were included in the study. Among all women, 1531 were less than 35 years old, and 715 were over or equal to 35 years old. Each age group was divided into three groups: low AMH, medium AMH and high AMH. The baseline characteristics by age and AMH groups are described in Table 1. In both the maternal age < 35 years and ≥ 35 years groups, women with low AMH were older than women with medium or high AMH, as expected. This led to some differences in the stimulation protocols chosen, fertilization methods used, number of oocytes retrieved, number of mature and normal fertilized oocytes and number of normal cleavages of 2PN.

**Table 1:** Baseline characteristics and IVF-ET data of the women in the different AMH and age groups

Characteristics	Age < 35 y (*n* = 1531)				Age ≥ 35 y (*n* = 715)			
	Low AMH (*n* = 324)	Medium AMH (*n* = 746)	High AMH (*n* = 461)	*P*- value	Low AMH (*n* = 272)	Medium AMH (*n* = 343)	High AMH (*n* = 100)	*P*- value
Age (years)	31 (20–34) ^a^	31 (20–34) ^b^	30 (20–34) ^c^	< 0.0001	37 (35–46) ^a^	37 (35–46) ^b^	36 (35–42) ^c^	< 0.0001
AMH (ng/ml)	1.10 (0.06–1.60) ^a^	2.70 (1.61–3.98) ^b^	5.22 (3.99–16.21) ^c^	< 0.0001	1.05 (0.02–1.60) ^a^	2.50 (1.61–3.91) ^b^	5.00 (4.00–14.30) ^c^	< 0.0001
BMI (kg/m^2^)	22.6 (20.7–25.4) ^a, b^	22.3 (16.0–33.3) ^b^	22.8 (15.6–36.1) ^a^	0.007	23.0 (16.0–33.2)	23.0 (16.5–35.8)	23.0 (17.2–33.9)	0.931
Previous conception [% (n/N)]	24.4 (79/324)	24.1 (180/746)	23.2 (107/461)	0.912	44.5 (121/272)	47.2 (162/343)	45.0 (45/100)	0.780
Duration of infertility (years)	4 (1–14)	4 (1–12)	4 (1–14)	0.636	4 (1–20)	4 (1–17)	5 (1–16)	0.377
Primary infertility [% (n/N)]	62.0 (201/324)	60.9 (454/746)	62.3 (287/461)	0.869	39.0 (106/272)	35.6 (122/343)	42.0 (42/100)	0.441
Main diagnosis				< 0.0001				< 0.0001
Ovulation disorder	6.2 (20/324) ^a^	7.5 (56/746) ^a^	26.5 (122/461) ^b^		2.6 (7/272) ^a^	7.6 (26/343) ^b^	19.0 (19/100) ^c^	
Tubal factor	42.3 (137/324) ^a, b^	48.1 (359/746) ^b^	37.7 (174/461) ^a^		41.5 (113/272)	41.4 (142/343)	46.0 (46/100)	
Male factor	17.3 (56/324) ^a^	23.3 (174/746) ^a, b^	24.5 (113/461) ^b^		15.8 (43/272)	18.1 (62/343)	17.0 (17/100)	
Endometriosis	15.7 (51/324) ^a^	10.7 (80/746) ^a^	5.2 (24/461) ^b^		12.9 (35/272)	10.8 (37/343)	7.0 (7/100)	
Unexplained	18.5 (60/324) ^a^	10.3 (77/746) ^b^	6.1 (28/461) ^c^		27.2 (74/272) ^a^	22.2 (76/343) ^a^	11.0 (11/100) ^b^	
Internal medicine diseases	8.6 (28/324)	7.6 (57/746)	6.1 (28/461)	0.372	4.8 (13/272)	8.5 (29/343)	6.0 (6/100)	0.186

*AMH* anti-Müllerian hormone, *BMI* body mass index

Continuous data are shown as the median (range) and were analysed using the Kruskal–Wallis test

Categorial variables are shown as percentages (number) and were analysed using the chi-square test

The superscripts including the same letter indicate that there was no significant difference among the women with different AMH levels

Regarding the in vitro embryo developmental process, as shown in Table 2, when the maternal age was < 35 years, the ratios of MII oocytes to total oocytes retrieved were not significantly different among the different AMH groups. However, when the maternal age was ≥ 35 years, the ratio of MII oocytes to total oocytes retrieved was significantly higher in the low-AMH group than in the high-AMH group. However, there were no statistically significant differences in the normal fertilization rate or cleavage rate. In the < 35-year age group, the rates of high-quality embryos on D3 in the low-AMH and high-AMH groups were higher than those in the medium-AMH group, but only the rates in the high-AMH and medium-AMH groups were significantly different.

**Table 2:** The characteristics of the IVF/ICSI treatments and outcomes of the women in the different AMH and age groups

Characteristics	Age < 35 y (*n* = 1531)				Age ≥ 35 y (*n* = 715)			
	Low AMH (*n* = 324)	Medium AMH (*n* = 746)	High AMH (*n* = 461)	*P*- value	Low AMH (*n* = 272)	Medium AMH (*n* = 343)	High AMH (*n* = 100)	*P*- value
Stimulation protocol				< 0.0001				< 0.0001
GnRH agonist	34.0 (110/324) ^a^	51.1 (381/746) ^b^	51.8 (239/461) ^b^		24.3 (66/272) ^a^	47.2 (162/343) ^b^	49.0 (49/100) ^b^	
GnRH antagonist	59.9 (194/324) ^a^	48.5 (362/746) ^b^	47.9 (221/461) ^b^		68.8 (187/272) ^a^	51.6 (177/343) ^b^	50.0 (50/100) ^b^	
Others	6.2 (20/324) ^a^	0.4 (3/746) ^b^	0.2 (1/461) ^b^		7.0 (19/272) ^a^	1.2 (4/343) ^b^	1.0 (1/100) ^a, b^	
Fertilization method				< 0.0001				0.028
IVF	58.0 (188/324) ^a^	49.7 (371/746) ^b^	44.3 (204/461) ^b^		58.1 (158/272)	53.1 (182/343)	51.0 (51/100)	
ICSI	41.7 (135/324) ^a,^	47.7 (356/746) ^a, b^	50.5 (233/461) ^a^		41.5 (113/272)	46.1 (158/343)	45.0 (45/100)	
IVF + ICSI	0.3 (1/324) ^a^	2.5 (19/746) ^b^	5.2 (24/461) ^c^		0.4 (1/272) ^a^	0.9 (3/343) ^a, b^	4.0 (4/100) ^b^	
Number of oocytes retrieved	7 (1–25) ^a^	12 (1–28) ^b^	15 (1–34) ^c^	< 0.0001	5 (1–23) ^a^	10 (1–25) ^b^	13 (4–28) ^c^	< 0.0001
Number of mature oocytes	5 (1–21) ^a^	10 (1–26) ^b^	12 (1–29) ^c^	< 0.0001	4 (1–19) ^a^	9 (1–23) ^b^	11 (2–25) ^c^	< 0.0001
Rate of MII (%)	84.9 (1922/2263)	83.1 (7516/9046)	83.1 (5690/6845)	0.092	86.9 (1351/1554) ^a^	84.6 (3052/3609) ^a, b^	83.6 (1174/1405) ^b^	0.026
Number of 2PN	4 (1–17) ^a^	7 (1–25) ^b^	9 (1–25) ^c^	< 0.0001	3 (0–17) ^a^	6 (1–16) ^b^	8 (2–19) ^c^	< 0.0001
Normal fertilization rate [% (n/N)]	70.9 (1454/2052)	70.7 (5734/8105)	70.7 (4298/6078)	0.992	71.1 (1011/1422)	68.7 (2248/3274)	70.1 (871/1242)	0.221
Normal cleavages of 2PN	4 (0–17) ^a^	7 (0–25) ^b^	9 (1–24) ^c^	< 0.0001	3 (0–17) ^a^	6 (1–16) ^b^	8 (2–19) ^c^	< 0.0001
Cleavage rate (%)	98.0 (1425/1454)	97.6 (5596/5734)	97.8 (4203/4298)	0.595	98.7 (998/1011)	98.8 (2220/2248)	97.9 (853/871)	0.202
Number of D3 high-quality embryos	1 (0–8) ^a^	2 (0–16) ^b^	3 (0–15) ^c^	< 0.0001	1 (0–14) ^a^	2 (0–11) ^b^	3 (0–16) ^c^	< 0.0001
High-quality embryo rate [% (n/N)]	39.3 (560/1425) ^a, b^	36.7 (2053/5596) ^b^	39.6 (1664/4203) ^a^	0.008	40.1 (400/998)	37.8 (840/2220)	38.7 (330/853)	0.480
Number of embryos transferred	2 (1–2) ^a^	2 (1–2) ^b^	1 (1–2) ^c^	< 0.0001	2 (1–3)	2 (1–3)	2 (1–3)	0.058
Number of embryos implanted	0 (0–2)	0 (0–2)	0 (0–2)	0.559	0 (0–2)	0 (0–2)	0 (0–2)	0.052
Implantation rate [% (n/N)]	34.5 (193/560)	35.3 (434/1230)	37.5 (247/658)	0.488	22.7 (107/471)	28.3 (167/590)	23.9 (38/159)	0.102
Pregnancy rate [% (n/N)]	46.9 (152/324) ^a^	46.0 (343/746) ^a^	44.7 (206/461) ^b^	0.818	34.2 (93/272) ^a^	43.1 (148/343) ^b^	33.0 (33/100) ^a^	0.038
Biochemical pregnancy rate [% (n/N)]	4.6 (15/324) ^a^	4.7 (35/746) ^b^	3.5 (16/461) ^a^	0.568	3.3 (9/272)	5.2 (18/343)	7.0 (7/100)	0.281
Early miscarriage rate [% (n/N)]	5.6 (18/324) ^a^	2.5 (19/746) ^b^	5.4 (25/461) ^a^	0.015	6.6 (18/272)	8.5 (29/343)	8.0 (8/100)	0.692
Ectopic pregnancy rate [% (n/N)]	2.2 (7/324) ^a^	2.1 (16/746) ^b^	1.3 (6/461) ^a^	0.536	1.8 (5/272)	2.0 (7/343)	1.0 (1/100)	0.866
Live birth rate [% (n/N)]	37.3 (121/324)	39.9 (298/746)	36.0 (166/461)	0.368	22.8 (62/272)	30.3 (104/343)	24.0 (24/100)	0.091

*AMH* anti-Müllerian hormone, *2PN* 2 pronuclear zygotes

Continuous data are shown as the median (range) and were analysed using the Kruskal–Wallis test

Categorial variables are shown as percentages (number) and were analysed using the chi-square test

The superscripts including the same letter indicate that there was no significant difference among the women with different AMH levels

**Fig. 2:**
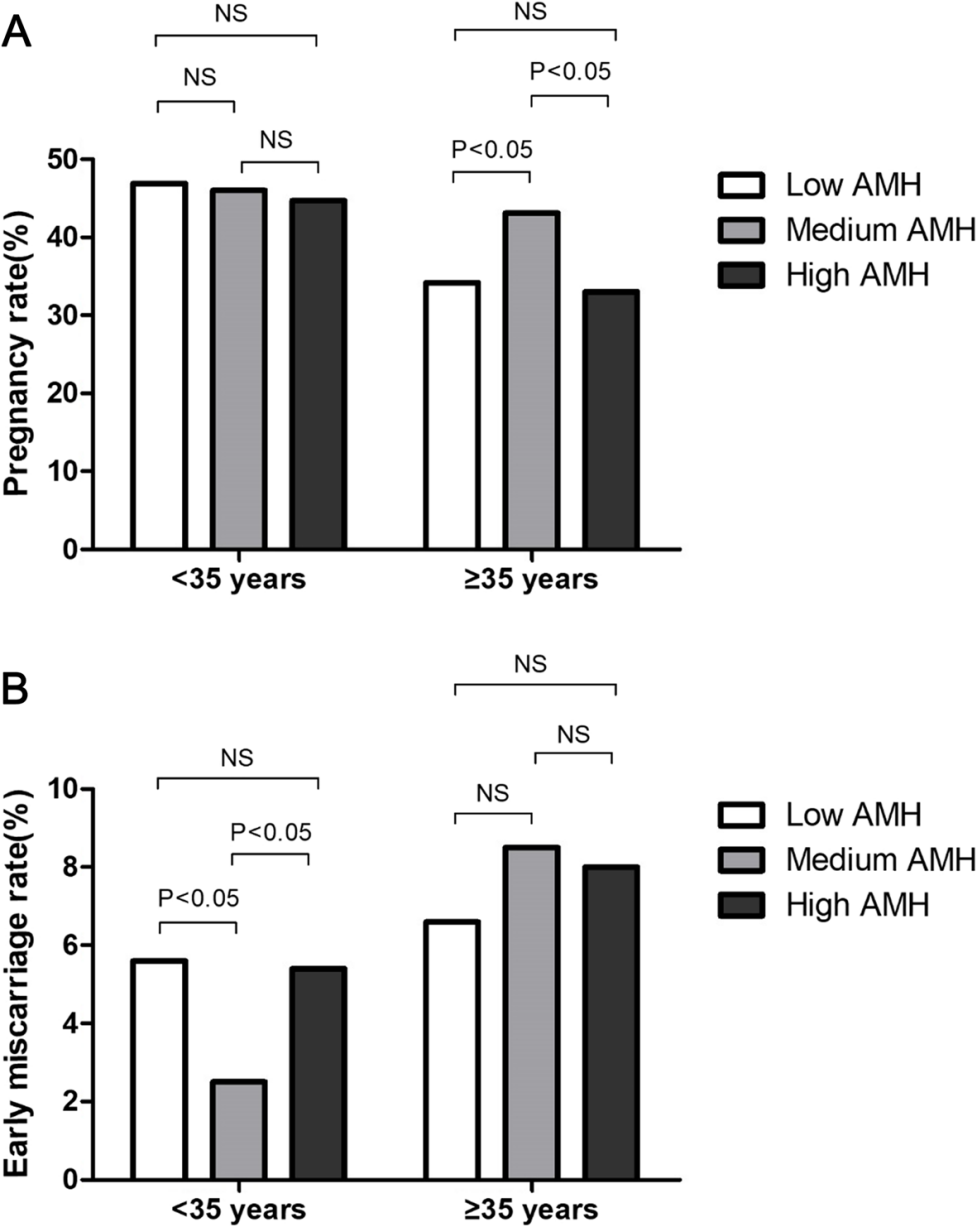
The unadjusted pregnancy rates and early miscarriage rates among the women in the different AMH and age groups. **A** χ2 testing showed that in the ≥ 35-years age group, the pregnancy rate was significantly higher in the medium (1.61–3.98 ng/ml) AMH group than that in the low (0.06–1.60 ng/ml) or high (3.99–20.20 ng/ml) AMH group. However, in the < 35-years age group, the pregnancy rate of the different AMH groups were not significantly different. **B** χ2 testing showed that in the < 35-years age group, the early miscarriage rate was significantly lower in the medium AMH group than in the low or high AMH group. However, in the ≥ 35-years age group, the early miscarriage rates of the different AMH groups were not significantly different

The clinical outcomes, including implantation rates, pregnancy rates, biochemical pregnancy rates, early miscarriage rates, ectopic pregnancy rates and live birth rates, in the different age and AMH groups are shown in Table 2. When women were ≥ 35 years old, the pregnancy rate was significantly higher in the medium-AMH group than in both the low-AMH and high-AMH groups. However, when women were < 35 years old, the pregnancy rates in the three AMH groups were not significantly different. We found that the early miscarriage rate was significantly lower in the medium-AMH group than in either the low-AMH or high-AMH group when women were < 35 years old (Fig. 2). The live birth rate was higher in the medium-AMH group than in either the low-AMH or high-AMH group, but the differences did not reach statistical significance. In addition, the implantation rates, biochemical pregnancy rates, ectopic pregnancy rates and live birth rates among the different age and AMH groups were not significantly different.

The associations of AMH categories with pregnancy rate and early miscarriage rate are shown in Tables 3 and 4. When women were ≥ 35 years old, women with low AMH had a significantly decreased risk for pregnancy compared with women with medium AMH (unadjusted OR 0.685, 95% CI 0.492 to 0.951), but after adjusting for the confounders, different AMH levels were not significantly associated with pregnancy (adjusted OR 0.717, 95% CI 0.491 to 1.048). When women were ≥ 35 years old, women with high AMH were not significantly associated with pregnancy compared with women with medium AMH (unadjusted OR 0.649, 95% CI 0.406 to 1.037), but after adjusting for the confounders, high AMH levels were associated with a significant decreased risk for pregnancy (adjusted OR 0.579 95% CI 0.352 to 0.953). However, after excluding women with PCOS, the association between high AMH levels and pregnancy disappeared (adjusted OR 0.621 95% CI 0.375 to 1.027). When women were < 35 years old, compared with medium AMH levels, neither low nor high AMH levels were associated with the pregnancy rate, even after adjusting for confounders (low AMH: adjusted OR 0.951, 95% CI 0.715 to 1.266; high AMH: adjusted OR 0.964, 95% CI 0.750 to 1.238). The results remained similar after excluding women with PCOS (low AMH: adjusted OR 0.952, 95% CI 0.715 to 1.268; high AMH: adjusted OR 0.983, 95% CI 0.762 to 1.269). When women were < 35 years old, women with low or high AMH levels had a significantly increased risk for early miscarriage compared with women with medium AMH levels (low AMH: unadjusted OR 2.251, 95% CI 1.165 to 4.348; high AMH: unadjusted OR 2.194, 95% CI 1.194 to 4.031). After adjusting for confounders, only women with high AMH levels had a significant increased early miscarriage risk (low AMH: adjusted OR 1.923, 95% CI 0.930 to 3.977; high AMH: adjusted OR 2.3826, 95% CI 1.246 to 4.553). Among women who were ≥ 35 years old, those with low or high AMH levels had no increase in their early miscarriage risk (low AMH: adjusted OR 0.661, 95% CI 0.335 to 1.304; high AMH: adjusted OR 1.168, 95% CI 0.489 to 2.789). The results remained similar after excluding women with PCOS among women aged < 35 years or ≥ 35 years.

**Table 3:** Crude and adjusted odds ratios (ORs) of different AMH levels for pregnancy rates among women aged < 35 years or ≥ 35 years

	Age < 35 y				Age ≥ 35 y			
	Unadjusted OR (95% CI)	*P*-value	Adjusted OR (95% CI)	*P*-value	Unadjusted OR (95% CI)	*P*-value	Adjusted OR (95% CI)	*P*-value
All women								
AMH								
Low AMH	1.038 (0.799–1.349)	0.778	0.951 (0.715–1.266)	0.732	0.685 (0.492–0.951)	0.024	0.717 (0.491–1.048)	0.086
Medium AMH	Reference				Reference			
High AMH	0.949 (0.752–1.199)	0.661	0.964 (0.750–1.238)	0.772	0.649 (0.406–1.037)	0.070	0.579 (0.352–0.953)	0.032
Women without PCOS								
AMH								
Low AMH	1.034 (0.796–1.343)	0.805	0.958 (0.719–1.278)	0.771	0.689 (0.496–0.958)	0.027	0.728 (0.497–1.065)	0.102
Medium AMH	Reference				Reference			
High AMH	0.977 (0.764–1.250)	0.855	0.968 (0.748–1.252)	0.802	0.696 (0.431–1.123)	0.137	0.621 (0.375–1.027)	0.064

Binary logistic regression analysis

ORs adjusted for maternal age, BMI, duration of infertility, main infertility diagnosis, history of internal medicine diseases, number of oocytes retrieved and high-quality embryo rate

**Table 4:** Crude and adjusted odds ratios of different AMH levels for early miscarriage rates among women aged < 35 years or ≥ 35 years

	Age < 35 y				Age ≥ 35 y			
	Unadjusted OR (95% CI)	*P*-value	Adjusted OR (95% CI)	*P*-value	Unadjusted OR (95% CI)	*P*-value	Adjusted OR (95% CI)	*P*-value
All women								
AMH								
Low AMH	2.251 (1.165–4.348)	0.016	1.923 (0.930–3.977)	0.078	0.767 (0.417–1.413)	0.395	0.661 (0.335–1.304)	0.233
Medium AMH	Reference				Reference			
High AMH	2.194 (1.194–4.031)	0.011	2.382 (1.246–4.553)	0.009	0.942 (0.416–2.130)	0.885	1.168 (0.489–2.789)	0.726
Women without PCOS								
AMH								
Low AMH	2.238 (1.159–4.324)	0.016	1.903 (0.917–3.948)	0.084	0.765 (0.415–1.409)	0.390	0.664 (0.337–1.309)	0.237
Medium AMH	Reference				Reference			
High AMH	2.269 (1.213–4.245)	0.010	2.493 (1.297–4.792)	0.006	1.016 (0.448–2.303)	0.970	1.184 (0.497–2.824)	0.703

Binary logistic regression analysis

ORs adjusted for maternal age, BMI, duration of infertility, main infertility diagnosis, history of internal medicine diseases, number of oocytes retrieved and high-quality embryo rate

## Discussion

This retrospective study aimed to explore whether serum AMH levels were independently related to some IVF outcomes. This study showed that high serum AMH levels (3.99–20.20 ng/ml) significantly increased the risk of early miscarriage compared with medium serum AMH levels (1.61–3.98 ng/ml) when women were < 35 years old, but when women were ≥ 35 years old early miscarriage did not show any significant association with different AMH levels.

Since maternal age has been clearly associated with IVF outcomes such as clinical pregnancy and spontaneous abortion rates [22–24], we divided patients into two groups based on maternal age: the < 35-year group and the ≥ 35-year group. In this study, in women < 35 years or ≥ 35 years of age, the number of oocytes retrieved, number of mature oocytes, number of 2PN, normal cleavages of 2PN and number of D3 high-quality embryos increased with increasing serum AMH levels. In our study, we found that women with high AMH levels had a lower rate of mature oocytes than women with low AMH levels. The differences were significant in women ≥ 35 years old but did not reach significance in women < 35 years old. We assume that the high serum AMH levels in women indicate more oocytes, which leads to less nutrition to individual oocytes to support maturation compared with women with low serum AMH levels. However, regardless of whether the woman was < 35 years or ≥ 35 years, the AMH level had no impact on the normal fertilization rate or the rate of normal cleavage, which indicated that the AMH level did not have an impact on the processes of fertilization and cleavage. It is widely accepted that age plays an essential role in the quality of oocytes [25]. Morin et al. [26] indicated that the lower chances of pregnancy in young women with low AMH levels undergoing IVF treatment might be due to the quantity of oocytes rather than the quality. The results were consistent with our study. In addition, women with high AMH levels had an increased rate of top embryo formation on D3 compared with women with medium AMH levels. The differences were significant in women < 35 years of age but not in women ≥ 35 years of age. The changes in the rate of top embryo formation between different AMH levels were inconsistent with the pregnancy outcomes, indicating that this might need further study with more cases included.

A healthy live birth is the ultimate goal of IVF treatment; therefore, pregnancy outcomes are the focus of our concern. In this study, we found that among women ≥ 35 years of age, those with medium (1.61–3.98 ng/ml) serum AMH levels had more pregnancies than women with low (0.06–1.60 ng/ml) or high (3.99–20.20 ng/ml) AMH levels in their first IVF or ICSI treatment. Among women of advanced maternal age, early miscarriage rates might be due mainly to poor-quality oocytes, which is usually caused by increasing age rather than AMH levels. That should be why in our study, early miscarriage rates among women ≥ 35 years of age did not show a significant association with different AMH levels. When women were < 35 years old, women with medium serum AMH levels had fewer early miscarriages than women with low or high AMH levels in their first IVF or ICSI treatment. The logistic regression analysis showed that high serum AMH levels significantly increased the early miscarriage rate compared with medium serum AMH levels when women were < 35 years old. The live birth rate in our study of women with medium AMH levels was higher than that of women with low or high AMH levels, but the differences did not reach statistical significance.

Similar to our study, Kostrzewa et al. [27] reported that in patients younger than 35 years with a spontaneous pregnancy, both low (< 1.1 ng/mL) and high (> 4.5 ng/mL) AMH concentrations significantly increased the risk of pregnancy loss in the first trimester. Gleicher et al. [28] built logistic regression models to predict IVF outcomes. They found that AMH at excessively high levels significantly increased the risk of miscarriage. Consistent with our research, Szafarowska et al. [29] found no significant differences between AMH concentration and pregnancy rates, and miscarriage rates were higher among women (age range 27–44 years) with AMH > 2.5 ng/mL than among those with AMH < 1 ng/mL or 1 ≤ AMH ≤ 2.5 ng/mL. However, the women of advanced maternal age included in their study might have affected the results, and this was not resolved. Gomezt et al. [30] reported that in patients older than 36 years, AMH could increase pregnancy rates, but in young patients, AMH levels could not influence pregnancy rates. In our study, among women ≥ 35 years of age high AMH levels also increased the pregnancy rates, but after adjusting for the possible confounding variables and excluding women with PCOS, the differences in pregnancy rates between different AMH levels were no longer evident.

AMH plays an important role in ovarian folliculogenesis. AMH inhibits primordial follicle recruitment and decreases the sensitivity of ovarian follicles for FSH [31]. Therefore, the higher concentrations of AMH lead to the greater inhibition of FSH, so that there may be a case where ovulation is stopped, such as in PCOS. The rates of pregnancy loss are higher among women with PCOS than among women without the syndrome [32, 33]. The abovementioned studies also showed similar pregnancy outcomes among women without PCOS but with high AMH levels [27–29]. Nilsson et al. [34] reported that AMH suppresses growth differentiation factor 9 (GDF9) and bone morphogenetic protein 15 (BMP15) expression. GDP9 and BMP15 are oocyte-secreted factors and play an important role in the process of follicular development and oocyte maturation [35, 36]. Mottershead et al. [37] confirmed that GDF9 and BMP15 are associated with oocyte quality. Li et al. [38] found that the expression levels of GDF9 and BMP15 mRNAs in cumulus granulosa cells in the group with high-quality embryos are significantly higher than those in the group without high-quality embryos (*P* < 0.05). In our study, the high AMH levels might be related to the inhibitory effect of AMH on GDP9 and BMP15. Decreasing GDP9 and BMP15 led to a decline in oocyte quality, which resulted in an increase in early miscarriage. Foetal aneuploidy is a major cause of early miscarriage. There are conflicting data regarding whether AMH is associated with an increase in aneuploid pregnancies. Shim et al. [39] found that low maternal AMH levels seemed to be associated with foetal aneuploidy in early pregnancy losses regardless of age. Some studies reported that maternal AMH was not a marker of foetal aneuploidy in ongoing pregnancies [40, 41]. However, in our study, the karyotypes of both the preimplantation embryos and abortion products were not analysed.

The results of studies on serum AMH levels and early miscarriage are conflicting. Contrary to our research, Zarek et al. [42]’s study found that among women who did not choose to undergo ART who had one or two previous pregnancy losses, neither low nor high AMH levels were associated with clinical loss compared with normal AMH levels. Pereira et al. [43]’s retrospective study of 1005 patients < 35 years of age with diminished ovarian reserve undergoing fresh IVF showed that AMH was not associated with the clinical pregnancy, spontaneous miscarriage or live birth rate. The limitations of our study included the fact that all the embryos transferred were only D3 embryos, and blastocysts were not included. Furthermore, frozen embryo transfer cycles were not analysed in this study. The aetiology of early miscarriage was unknown, and the karyotypes of the early miscarriage embryos were not detected. In addition, research that incorporates more patients would provide more information on the associations between AMH and early miscarriage.

In conclusion, among women < 35 years old, those with high (3.99–20.20 ng/ml) AMH levels had a higher likelihood of early miscarriage in the first IVF/ICSI fresh embryo transfer cycles than women with medium (1.61–3.98 ng/ml) AMH levels. These results suggest that AMH may play a part in predicting the competence of oocytes. Physicians should better exploit the value of AMH to provide more individualized consultation and predict IVF-ET outcomes for patients of different ages.

## Data Availability

The data used or analysed during the current study are included within the article. The datasets are not publicly available due to the hospital policy and personal privacy, but are available from the corresponding author on reasonable request.

## Abbreviations

AMH: Anti-Müllerian hormone
IVF/ICSI: In vitro fertilization/intracytoplasmic sperm injection
ET: Embryo transfer
BMI: Body mass index
PCOS: Polycystic ovary syndrome
GnRH: Gonadotrophin-releasing hormone
hCG: Human chorionic gonadotropin
2PN: 2 Pronuclear zygotes
OR: Odds ratio
